# Single-cell and spatial transcriptomics in intrahepatic cholangiocarcinoma: spatial immune ecosystems, metabolic remodeling, and immunotherapeutic implications

**DOI:** 10.3389/fimmu.2026.1918458

**Published:** 2026-10-20

**Authors:** Yanan Yan, Tianhao Teng, Xinying Luo, Jingfeng Liu, Jianmin Wang

**Affiliations:** 1 Innovation Center for Cancer Research, Clinical Oncology School of Fujian Medical University, Fujian Cancer Hospital, Fuzhou, China; 2 Fujian Key Laboratory of Advanced Technology for Cancer Screening and Early Diagnosis, Fujian Cancer Hospital, Fuzhou, China; 3 NHC Key Laboratory of Cancer Metabolism, Fujian Cancer Hospital, Fuzhou, China; 4 Department of Hepatopancreatobiliary Surgery, Clinical Oncology School of Fujian Medical University, Fujian Cancer Hospital, Fuzhou, China

**Keywords:** intrahepatic cholangiocarcinoma, single-cell RNA sequencing, spatial transcriptomics, tumor immune microenvironment, immunotherapy

## Abstract

Intrahepatic cholangiocarcinoma (iCCA) is an aggressive liver malignancy characterized by pronounced cellular, spatial, and metabolic heterogeneity. Single-cell RNA sequencing (scRNA-seq) and spatial transcriptomics (ST) are beginning to resolve this complexity by linking malignant, immune, stromal, and metabolic states to their tissue context. This review synthesizes recent single-cell and spatial studies of iCCA across four spatial contexts: adaptive immune regions containing tertiary lymphoid structures (TLSs), barriers formed by stromal and myeloid cells at the invasive front, myeloid niches shaped by hypoxia, and metastatic ecosystems involving immune and stromal cells. These niches connect tumor heterogeneity with immune regulation, stromal remodeling, metabolic adaptation, and therapeutic vulnerability. We also evaluate how crosstalk between metabolism and immunity shapes macrophage polarization, T-cell dysfunction, and treatment response, while distinguishing computationally inferred interactions from functionally supported mechanisms. The field is therefore moving from cataloging additional cell states and spatial niches toward establishing which ecosystem features are reproducible across cohorts, mechanistically supported, clinically measurable, and informative for treatment response. Current evidence supports a view of iCCA as a spatially organized and metabolically regulated tumor ecosystem that can guide biomarker development and rational immunotherapeutic strategies.

## 1 Introduction

Intrahepatic cholangiocarcinoma (iCCA) is a primary liver malignancy with a steadily rising global incidence (1). Its pathogenesis is multifactorial, involving liver fibrosis, bile duct stones, and viral infections. Most patients present with intermediate- or advanced-stage disease at diagnosis, and fewer than 30% are eligible for surgical resection. Responses to radiotherapy, chemotherapy, and conventional targeted agents remain limited (2). Even with the introduction of immune checkpoint inhibitors, the 5-year overall survival rate for iCCA remains below 20% (3). Profound intertumoral and intratumoral heterogeneity is a defining feature of iCCA. Diverse malignant subclones, immune infiltrates, and stromal elements collectively shape a dynamic, treatment-refractory tumor microenvironment (TME) (4). Traditional bulk transcriptomic profiling has yielded valuable molecular classifications but is inherently constrained by an averaging effect. This masks the gene expression signatures of rare cell populations and removes the spatial context of cellular interactions (5).

The emergence of single-cell RNA sequencing (scRNA-seq) has transformed our ability to dissect the cellular architecture of complex tissues. By profiling transcriptomes at single-cell resolution, scRNA-seq enables precise identification of cell subtypes, differentiation trajectories, and functional states within the iCCA TME (6). However, the enzymatic and mechanical dissociation required by scRNA-seq destroys native tissue architecture. This removes critical spatial information about cell–cell communication and niche-specific biology. Spatial transcriptomics (ST) technologies address this gap by mapping gene expression patterns onto intact tissue coordinates. They preserve the spatial context needed to understand how TME organization supports tumor growth and immune evasion (7).

Several recent reviews have summarized how single-cell sequencing has revealed cellular heterogeneity and tumor ecosystem complexity in cholangiocarcinoma (4, 5, 8). Building on these previous syntheses, we integrate scRNA-seq and ST evidence to examine iCCA heterogeneity in relation to spatial immune organization, metabolic remodeling, and therapeutic vulnerability. Integrated analyses can help infer the locations of scRNA-seq-defined cell states and their arrangement within pathological regions, including the tumor core and invasive front. They can also identify candidate interactions that may warrant further validation. This spatially informed perspective is particularly relevant to iCCA, a highly desmoplastic malignancy with marked immune and stromal heterogeneity (9). Hypoxic regions and metastatic niches add further spatial complexity within individual lesions.

In this review, we synthesize recent single-cell and spatial studies of iCCA from an ecosystem-centered perspective. The complementary capabilities of scRNA-seq and ST are considered in relation to how cellular states are positioned within spatially defined tumor ecosystems. We examine the organization of tumor, immune, stromal, and metabolic programs across four spatial contexts. These comprise tertiary lymphoid structure (TLS)-associated immune regions, stromal–myeloid barriers at the invasive front, hypoxia-associated myeloid niches, and metastatic ecosystems shaped by immune and stromal cells. These spatial and metabolic features are further linked to biomarker development and the biological rationale for current and emerging immunotherapeutic strategies. Representative scRNA-seq and ST studies of iCCA discussed in this review are summarized in **Supplementary Table 1**.

We conducted a narrative literature search and synthesis. PubMed, Web of Science, and Google Scholar were searched from database inception through August 2026. Search terms included combinations of “intrahepatic cholangiocarcinoma,” “cholangiocarcinoma,” “single-cell RNA sequencing,” “spatial transcriptomics,” “tumor microenvironment,” “immunotherapy,” “macrophage,” “cancer-associated fibroblast,” “tertiary lymphoid structure,” “metabolism,” and “ligand–receptor interaction.” Studies were prioritized when they specifically investigated iCCA using single-cell sequencing, spatial transcriptomic or proteomic approaches, multi-omic analyses, functional experiments, or clinically annotated datasets and provided information relevant to the tumor microenvironment or therapeutic response. Evidence from broader biliary tract cancer or liver cancer studies was included selectively when iCCA-specific data were limited or when such studies provided relevant mechanistic or therapeutic context. Findings derived from these broader disease settings were distinguished from and interpreted separately from iCCA-specific evidence.

## 2 Single-cell RNA sequencing and spatial transcriptomics

scRNA-seq and ST provide complementary approaches to dissecting the TME of iCCA. Bulk transcriptomic profiling remains useful for defining dominant molecular programs, but it inevitably averages signals from tumor, immune, stromal, and other cellular compartments. By contrast, scRNA-seq enables transcriptome-wide profiling of individual cells, thereby resolving rare populations, transitional states, and lineage-associated programs that are obscured in bulk analyses (10). In iCCA, scRNA-seq has helped characterize malignant epithelial heterogeneity, immunosuppressive cell states, stromal remodeling, and therapy-associated cellular programs (4, 5, 11). However, scRNA-seq also removes cells from their tissue context. Dissociation can alter transcriptional programs and cause selective loss of fragile or spatially embedded cells. It also erases anatomical relationships that shape interactions among tumor, immune, and stromal compartments *in situ*. Therefore, scRNA-seq findings provide high-resolution cellular evidence for generating mechanistic hypotheses. They do not directly establish spatial organization or functional causality. ST complements scRNA-seq by preserving gene expression within the anatomical architecture of tissue sections (12).

Current ST approaches can be broadly divided into imaging-based and sequencing-based strategies. Imaging-based ST approaches, including MERFISH, Xenium, CosMx, STARmap, and seqFISH+, detect predefined transcripts directly in tissue sections through multiplexed hybridization or *in situ* sequencing (13–17). These platforms can provide high spatial precision and, when tissue quality and cell segmentation are adequate, can localize transcripts at subcellular or near-single-cell scales (7). This makes them particularly useful for examining spatially organized structures such as tumor borders, immune aggregates, macrophage–fibroblast interfaces, and tertiary lymphoid structures. Their main limitation is that most assays rely on targeted gene panels, requiring prior selection of transcripts of interest and potentially missing unexpected pathways or rare cellular programs. Their cell-level accuracy also depends on probe performance, image quality, tissue preservation, and reliable transcript-to-cell assignment.

Sequencing-based ST uses spatially barcoded capture or related *in situ* barcoding strategies to link RNA molecules to tissue coordinates before sequencing. Representative approaches include Visium-derived platforms, Stereo-seq, Slide-seqV2, and related high-density barcoding methods (18–21). Compared with targeted imaging-based methods, sequencing-based ST generally offers broader transcriptomic coverage and is well suited for exploratory profiling across larger tissue regions (22). This breadth is important in iCCA, where tumor nests, desmoplastic stroma, invasive fronts, vascular regions, and immune-rich areas may coexist within the same lesion. Nevertheless, sequencing-based ST usually involves a trade-off between transcriptomic breadth and cellular precision. Spatial bins or capture units may contain mixed RNA signals from multiple neighboring cells, partial cellular fragments, extracellular RNA, or closely apposed immune and stromal cells. Thus, even platforms with dense spatial sampling should not automatically be interpreted as producing experimentally resolved single-cell transcriptomes.

The distinction between spatial resolution and true single-cell resolution is essential for interpreting ST data in iCCA. Spatial resolution refers to the physical scale at which transcripts are localized or captured within a tissue section. True single-cell resolution requires reliable assignment of detected transcripts to individual cells. This usually depends on accurate nuclear or membrane segmentation and transcript-to-cell allocation. Small capture spots, dense spatial barcodes, or subcellular-scale sampling do not necessarily mean that each measured signal derives from one intact cell. Conversely, imaging-based assays can localize transcripts with high positional accuracy, but inaccurate segmentation may still misassign transcripts to adjacent cells, especially in densely cellular tumor regions. These caveats are particularly relevant for iCCA because glandular tumor structures, dense desmoplastic stroma, and heterogeneous immune infiltration complicate cell-boundary delineation.

The complementary strengths and limitations of scRNA-seq and ST have motivated computational integration strategies that combine dissociated single-cell references with spatially resolved tissue profiles (23, 24). For the purposes of this review, these approaches can be broadly grouped into deconvolution and spatial mapping. Deconvolution methods use scRNA-seq reference data to estimate the proportions of cell types or cell states within spatial locations, thereby reconstructing the likely cellular composition of histological regions (25). Spatial mapping approaches project cell types or transcriptional states defined by scRNA-seq onto tissue coordinates, enabling inferred localization of cellular programs within tissue architecture (14). These methods are powerful for linking cell identity to anatomical context, but they remain inferential and model dependent. Their accuracy depends on reference atlas completeness, platform compatibility, tissue sampling, segmentation quality, batch effects, and the transcriptional similarity of closely related cell states. Rare populations absent from the reference atlas, dissociation-induced transcriptional changes, or imputation of unmeasured genes can further affect the apparent spatial distribution of cell types and pathways.

Accordingly, integrated scRNA-seq and ST analyses provide spatially informed inference rather than direct evidence of single-cell spatial organization or functional signaling. Spatial colocalization and computational ligand–receptor compatibility can identify candidate interactions and define the cellular contexts in which they may occur. Histology, immunostaining, and multiplex imaging further resolve the localization and proximity of the implicated cell populations and molecules within tissue architecture. Functional perturbation, including ligand or receptor blockade and genetic manipulation, can then determine whether disruption of a proposed interaction alters the predicted cellular or phenotypic response, thereby strengthening evidence for a mechanistic relationship. Clinical association studies add a complementary translational dimension by linking candidate cell states, spatial features, or interaction programs to prognosis and treatment response, although such associations alone do not establish functional signaling. Integration of cellular profiling, spatial localization, functional perturbation, and clinical association extends the study of iCCA beyond a descriptive inventory of tumor, immune, and stromal cell types toward a spatially organized ecosystem in which cellular states, tissue niches, and intercellular interactions collectively contribute to tumor progression and therapeutic response.

## 3 The spatially organized immune ecosystem in iCCA

Spatial profiling has shifted the view of the iCCA tumor microenvironment from a collection of immune-cell populations to a spatially organized ecosystem. Within this ecosystem, tumor cells, stromal components, and immune populations interact in anatomically defined niches. This organization is particularly relevant in iCCA, a highly desmoplastic malignancy characterized by extensive fibroblast activation, macrophage enrichment, vascular remodeling, and marked heterogeneity in immune cell distribution. Rather than being uniformly immunosuppressive or immune-active, iCCA tumors contain spatial compartments with distinct biological functions. These include TLS-associated adaptive immune regions, tumor–stromal interfaces at the invasive front, hypoxia-associated myeloid niches, and myeloid–tumor ecosystems at primary and metastatic sites. These spatial architectures integrate tumor cell states, stromal organization, and immune cell programs across the iCCA microenvironment. This integration influences immune surveillance, tumor progression, and therapeutic vulnerability.

### 3.1 Tertiary lymphoid structures: location- and context-dependent adaptive immunity

TLSs are ectopic lymphoid aggregates that develop in non-lymphoid tissues and can support B-cell maturation, antibody production, and local antigen-driven immune responses (26–28). In iCCA, TLSs are clinically meaningful not only because they are present, but because their anatomical distribution and tissue context shape their prognostic associations. Ding et al. reported that intratumoral TLSs were associated with favorable survival, whereas a high peritumoral TLS score was associated with poorer outcomes. Integrating these two spatial scores separated tumors into immune classes with distinct prognoses (29). Doi et al. subsequently associated high densities of both intratumoral and peritumoral TLSs with prolonged survival in large-duct-type iCCA. Peritumoral TLSs retained prognostic significance within this histologic subtype in their analysis (30). These findings place TLS spatial distribution at the center of iCCA immune stratification. The biological meaning of peritumoral TLSs may depend on histologic subtype, stromal context, and the assessment of TLS maturity and location.

The cellular composition reported by Ding et al. provides one explanation for this context dependence. Intratumoral TLSs in iCCA are enriched for CD4^+^ BCL6^+^ T follicular helper (Tfh) cells, consistent with B-cell maturation and humoral immune support. CD4^+^ FOXP3^+^ regulatory T cells (Tregs) are also present in these structures. Their frequency within intratumoral TLSs correlates with the poor-prognosis peritumoral TLS score rather than the favorable intratumoral TLS score (29). This distinction argues against interpreting Treg abundance in isolation. In organized intratumoral TLSs, Tregs may coexist with Tfh and B-cell structures in an immune-active environment. By contrast, Treg-rich peritumoral lymphoid aggregates in other settings may reflect a less permissive stromal or inflammatory architecture. The favorable association of peritumoral TLSs in large-duct-type iCCA further emphasizes that TLS function is shaped by tissue context, maturity, and neighboring stromal or vascular features, rather than by location alone (30).

B-cell profiling adds a functional layer to this TLS-centered view. Multimodal single-cell analysis showed that B cells were enriched in adjacent tumor-free tissue, where mature TLSs were observed and associated with favorable prognosis (31). By contrast, tumor-infiltrating B cells were relatively scarce and showed reduced effector differentiation together with increased expression of immunoregulatory factors, including IL-10 and TGF-β. Ex vivo coculture experiments showed that exposure to tumor cells or cancer-associated fibroblasts (CAFs) impaired B-cell differentiation and reduced BAFFR expression. IL-6 and TGF-β mediated this dysfunctional state, and their combined blockade restored B-cell activation and differentiation (31). In the same study, elevated circulating BAFFR^+^ B cell frequencies and hyperexpanded B-cell clonotypes were associated with improved responses to chemoimmunotherapy. These findings indicate an association between the functional state of B cells and treatment response.

Together, these studies suggest that TLS abundance alone is an incomplete descriptor of adaptive immune organization in iCCA. TLS location and maturation status, the organization of Tfh cells and B cells, and the functional competence of B cells appear more informative than a simple TLS-positive versus TLS-negative classification. A preclinical iCCA study supports this interpretation: VEGF blockade potentiated combined anti-CTLA-4 and anti-PD-L1 therapy through a BAFF- and IL-12-dependent immune mechanism. This mechanism included both proinflammatory B-cell responses and TLS formation (32). Future iCCA studies could assess TLS location and maturation alongside the frequency of BAFFR^+^ B cells, the clonality of B cells, and treatment response. This could move TLS assessment beyond a static histologic feature toward spatially informed immune stratification.

### 3.2 The invasive front: coordinated remodeling of tumor, stromal, and immune compartments

The tumor invasive front forms a transitional interface between malignant parenchyma and surrounding liver tissue. Here, tumor cells, CAFs, endothelial cells, macrophages, and lymphocytes are spatially organized within a distinct microenvironmental compartment. The invasive front may function as a dynamic remodeling interface in which tumor cell plasticity, stromal remodeling, and immune reorganization converge. Spatial studies of liver cancer and iCCA indicate that this region integrates tumor-cell adaptation with extracellular matrix remodeling, vascular changes, and altered immune cell distribution.

Malignant cells at the invasive border display transcriptional states distinct from those in deeper tumor regions. Based on Stereo-seq, Wu et al. identified an invasive zone in human liver cancer, including iCCA samples. This zone showed enhanced programs associated with epithelial–mesenchymal transition, hypoxia, angiogenesis, and lipid metabolism, with lower proliferative activity than deeper tumor regions (33). In an iCCA-specific study, Li et al. identified malignant cells near POSTN^+^ FAP^+^ CAFs, endothelial cells, and SPP1^+^ tumor-associated macrophages (TAMs). These malignant cells exhibited proliferative and mesenchymal features, including increased expression of MKI67, TOP2A, and mesenchymal-associated genes (34). The invasive margin contains heterogeneous malignant cell states associated with migration, metabolic adaptation, and stromal interaction. These states provide a cellular context for subsequent immune and stromal remodeling.

CAF organization is a defining feature of invasive-front architecture. Spatial and single-cell analyses have shown preferential enrichment of POSTN^+^ FAP^+^ matrix CAFs at invasive or leading-edge regions, where they contribute to extracellular matrix deposition and stromal organization (33, 34). CD146^+^ vascular CAFs represent another fibroblast state associated with the tumor core and microvascular regions. These CAFs interact with tumor cells through IL-6/IL-6R signaling (35). In addition to these spatially defined CAF populations, mechanistic studies have revealed how tumor-intrinsic programs can promote fibroblast activation. ARID1A was reported to stabilize NICD1 by suppressing AMPK-dependent autophagy and lysosomal degradation, sustaining Notch signaling and increasing TLL1 expression. Tumor-derived TLL1 activated hepatic stellate cells, whereas TLL1 depletion reduced CAF accumulation, collagen deposition, and tumor progression (36). Spatial localization of these CAF states, coupled with mechanistic evidence, suggests that tumor cells can reinforce fibroblast-rich stromal remodeling through both local positioning and tumor-derived activating signals.

Myeloid remodeling occurs alongside CAF accumulation and further shapes the ecosystem at the invasive front. Li et al. used pseudotime analysis to infer a macrophage-state trajectory from FOLR2^+^ TAMs toward SPP1^+^ TAMs and subsequently MKI67^+^ proliferative states. This inferred trajectory was accompanied by downregulation of phagocytic programs and upregulation of fatty-acid metabolism, hypoxia, and glycolysis-related signatures (34). The same study identified potential roles for CD8^+^ mucosal-associated invariant T (MAIT) cells in macrophage recruitment through CCL5–CCR1 and CSF1–CSF1R signaling. Antitumor programs in MAIT cells showed a graded reduction across the normal liver, invasive front, and tumor core. In parallel, POSTN^+^ FAP^+^ CAFs, SPP1^+^ TAMs, and endothelial cells form a spatially organized cellular network at the leading edge (34). Broader liver cancer analyses further showed that FAP^+^ CAFs and TAMs can accumulate within extracellular matrix-rich regions associated with immune exclusion. Coupling between SPP1^+^ TAMs and FAP^+^ CAFs was particularly evident in iCCA (37). These findings position CAFs, macrophages, and endothelial cells as interconnected components of the niche at the invasive front rather than isolated stromal populations.

The combined remodeling of tumor cells, fibroblasts, and myeloid populations influences lymphocyte localization and function within the invasive margin. CAF-rich extracellular matrix organization can restrict immune cell access, while macrophage-derived inflammatory and immunoregulatory signals further shape local T-cell states. IL-6/STAT3 signaling is a recurring inflammatory axis within this environment. IL-6 from vascular and inflammatory CAFs can promote tumor cell stemness, while tumor-derived signals such as exosomal miR-9-5p enhance CAF IL-6 production (35). In KRAS-mutant iCCA, IL-6/STAT3 signaling contributes to immunosuppressive neutrophil programming. IL-6 from inflammatory CAFs has also been associated with the accumulation of myeloid-derived suppressor cells and Tregs (35, 38, 39). Matrix remodeling, macrophage accumulation, and inflammatory signaling converge to create a spatial environment in which cytotoxic lymphocyte penetration and function are reduced.

The invasive front is therefore a niche in which tumor, stromal, and immune remodeling is coordinated with restricted cytotoxic lymphocyte access and function.

### 3.3 Hypoxia-associated myeloid niches: metabolic remodeling and immune polarization

Hypoxia shapes both the stromal and immune architecture of iCCA and affects multiple cellular compartments. Recent studies have identified hypoxia-responsive macrophage programs in primary tumors and intrahepatic metastatic lesions, linking low-oxygen stress to immune polarization, T cell regulation, and tumor cell invasion (40–42). These programs vary by compartment: hypoxia can support immunoregulatory organization of myeloid cells and Tregs in primary lesions and pro-invasive signaling between macrophages and tumor cells in metastatic settings.

Using scRNA-seq, Liang et al. characterized APOE^+^ TAMs as a hypoxia-responsive macrophage subset with metabolic and immunoregulatory properties in iCCA. Hypoxia activated the HIF1A–PPARγ–CD36 axis, increased PPARγ-dependent fatty acid oxidation, and promoted a lipid-oxidative, immunoregulatory state in APOE^+^ TAMs (42). Cell–cell communication analysis nominated CCL3–CCR5 as a candidate interaction between APOE^+^ TAMs and Tregs. ST and multiplex immunofluorescence supported the close spatial proximity of these populations within malignant lesions. In the same dataset, CXCL13^+^ precursor-exhausted CD8^+^ T cells represented a distinct state with high ENTPD1 and ITGAE expression. These cells showed greater cytotoxic activity and proliferative capacity than terminally exhausted CD8^+^ T cells, and their abundance correlated with improved survival (42). This population of T cells represents an additional component of the hypoxia-associated immune landscape. It indicates that suppressive organization of myeloid cells and Tregs can coexist with CD8^+^ T-cell states that retain functional potential.

In a complementary analysis of intrahepatic metastasis, Lin et al. identified ANGPTL4^+^ TAMs and hypoxia-responsive malignant epithelial cells enriched in metastatic lesions (41). Hypoxic stress increased CEBPB expression in macrophages, and CEBPB directly activated ANGPTL4 transcription. Macrophage-derived ANGPTL4 engaged integrin α5β1 on iCCA cells and activated the FAK/Src/GSK3β/β-catenin cascade, promoting β-catenin nuclear translocation and epithelial-mesenchymal remodeling. Functional *in vitro* assays and mouse metastasis models showed that ANGPTL4 enhanced migration, invasion, and liver metastasis, whereas ANGPTL4 blockade attenuated these phenotypes. Elevated ANGPTL4 levels in serum and tumor tissue also correlated with tumor burden and intrahepatic metastasis (41). This axis connects hypoxic macrophage adaptation to metastatic competence and supports the translational relevance of ANGPTL4 as a candidate circulating biomarker and therapeutic target.

APOE^+^ TAMs and ANGPTL4^+^ TAMs therefore illustrate two context-specific forms of hypoxia-associated myeloid remodeling. APOE^+^ TAMs are linked primarily to metabolic polarization and Treg enrichment within malignant lesions, whereas ANGPTL4^+^ TAMs are characterized in intrahepatic metastasis and directly support tumor cell invasion (41, 42). Hypoxia acts as a recurring selective pressure, but the resulting immune architecture depends on the tumor compartment, macrophage state, and neighboring malignant cell program.

### 3.4 Immunosuppressive niches involving myeloid and tumor cells across primary and metastatic compartments

Interactions between myeloid and tumor cells shape immune suppression across primary and metastatic compartments of iCCA. Across these anatomical settings, tumor cells interact with macrophages, fibroblasts, neutrophils, endothelial cells, and lymphocytes to establish spatially organized niches. These niches influence tumor cell plasticity, immune surveillance, and metastatic adaptation. They reflect compartment-specific interactions between malignant cell states and the surrounding immune and stromal environment.

Within primary tumors, colocalization of macrophages and tumor cells represents an important form of immunosuppressive organization. Fan et al. identified a macrophage-enriched infiltration pattern characterized by spatial colocalization of MARCO^+^ TAMs and CTSE^+^ tumor cells in iCCA (43). This cellular configuration was associated with metastatic behavior, reduced local immune activity, and unfavorable prognosis. Multiplex immunohistochemistry (mIHC) confirmed the spatial proximity of MARCO^+^ TAMs and CTSE^+^ tumor cells. Within this microenvironment, cell–cell communication analysis highlighted galectin signaling, including an LGALS9–CD44 ligand–receptor axis (43). Regions containing MARCO^+^ TAMs and CTSE^+^ tumor cells therefore constitute a macrophage-enriched niche in which myeloid signals and tumor cell programs converge.

Macrophages also contribute to tumor cell plasticity through reciprocal communication with tumor cells. Ruan et al. identified a macrophage-associated dedifferentiation and proliferation program in intratumoral iCCA (44). Malignant cell dedifferentiation and proliferation represented related but distinct states, with dedifferentiated cells characterized by KRT18 and TM4SF4 downregulation and proliferative cells marked by MKI67 and TOP2A expression. NicheNet analysis identified TGFB1 from SPP1^+^ TAMs and CXCL12 from resident macrophages as candidate signals associated with malignant cell state transitions. Spatial and functional observations supported the TGFB1–YAP1 axis in this setting. YAP1^+^ malignant cells localized near TGFB1^+^ CD68^+^ macrophages, and macrophage depletion reduced YAP1-positive dedifferentiated malignant cells in mice. Additional reciprocal crosstalk was predicted between malignant cells and myeloid populations. Macrophage-derived HMGB2 was nominated as a signal driving tumor cell proliferation, whereas IL-18 secreted by tumor cells was linked to the induction of LILRB1/LILRB2 expression in macrophages (44).

Metastatic lesions contain additional spatial niches in which malignant cell states reshape vascular and immune compartments. Using integrated scRNA-seq and ST, Xun et al. characterized spatiotemporal microenvironmental remodeling in iCCA liver metastases and identified a COL3A1^+^ epithelial subset with hybrid epithelial-mesenchymal features and enhanced invasive capacity (45). This metastatic epithelial state was associated with distinct microenvironmental interactions across dissemination-associated states. In inferred invasion-associated states, COL3A1^+^ epithelial cells were associated with endothelial remodeling, and cell–cell communication analysis identified JAG1–NOTCH3 as a candidate interaction with endothelial cells. Functional experiments independently demonstrated that COL3A1^+^ epithelial cells promote endothelial-to-mesenchymal transition and tumor cell invasion. In inferred colonization-associated states, COL3A1^+^ epithelial cells recruited VEGFA^+^ CCL4^+^ neutrophils. Functional analyses showed that these neutrophils generate neutrophil extracellular traps (NETs), which may reduce cytotoxic immune surveillance and support metastatic establishment (45). This study expands the metastatic immune niche beyond macrophage-centered models by showing how malignant cell states can organize vascular and neutrophil-associated immune environments in liver metastases.

Lymph node metastasis is another specialized immune and stromal compartment in iCCA. Li et al. characterized extensive remodeling of the nodal microenvironment and identified SAA1^+^ tumor cells and CD36^+^ lipid-metabolic TAMs as key populations involved in metastatic niche organization (46). Cell–cell communication analysis highlighted the SAA1–CD36 ligand–receptor axis, while mIHC demonstrated spatial proximity between SAA1^+^ tumor cells and CD36^+^ macrophages. *In vitro* inhibition of SAA1 or CD36 reduced tumor cell migration and invasion and decreased N-cadherin, Vimentin, and SMAD3 expression, linking this axis to metastatic tumor cell plasticity. In the same nodal ecosystem, CD36^+^ TAMs showed spatial association with exhausted CD8^+^ T cells and Tregs. THBS1^+^ myofibroblastic CAFs (myCAFs) were also enriched and associated with matrix remodeling and macrophage recruitment (46). The resulting lymph-node ecosystem is characterized by coordinated tumor, fibroblast, macrophage, and dysfunctional T-cell states that support metastatic adaptation.

### 3.5 An integrated spatial model of the iCCA immune ecosystem

Spatial studies indicate that the iCCA immune microenvironment is organized into interacting tissue modules. These include TLS-rich adaptive immune regions, stromal–myeloid interfaces at the invasive front, hypoxia-associated niches, and primary or metastatic myeloid–tumor ecosystems. These compartments link malignant cell states, stromal organization, and immune regulation across anatomical contexts. Cross-study support is strongest for broad patterns of TLS organization and stromal–myeloid remodeling, whereas several site-specific cellular programs have so far been described in narrower biological contexts.

A central feature of this ecosystem is the context dependence of immune and stromal cell states. SPP1^+^ TAMs participate in remodeling at the invasive front, APOE^+^ TAMs are linked to hypoxia-associated immunoregulatory programs, and CD36^+^ macrophages characterize metastatic lymph-node environments. Trajectory analysis in Li et al.’s dataset suggested transitions among selected macrophage states. However, current evidence does not support placing all macrophage populations identified across iCCA studies along a single differentiation continuum. Instead, SPP1^+^, MARCO^+^, APOE^+^, CD5L^+^, FOLR2^+^, and CD36^+^ populations may partly represent context-dependent transcriptional states shaped by distinct anatomical and metabolic niches. CAF programs show a similar dependence on spatial context. POSTN^+^ FAP^+^ CAFs are preferentially associated with invasive front regions, whereas CD146^+^ vascular CAFs are enriched in tumor core and microvascular compartments. Although these populations have been described using different subtype labels across studies, they converge functionally on extracellular matrix remodeling, vascular regulation, and immune cell exclusion. T-cell states are likewise spatially structured, with effector, precursor-exhausted, terminally exhausted, and MAIT cell programs occupying distinct immune niches and exhibiting different degrees of antitumor activity. Immune and stromal cell states in iCCA therefore reflect both lineage characteristics and the anatomical and cellular contexts in which they occur.

Reciprocal interactions among malignant, immune, stromal, and vascular compartments generate spatially heterogeneous immune states within the same tumor. This interdependence helps explain why targeting a single immune pathway may be insufficient in tumors composed of multiple interacting niches.

This spatial organization is also beginning to inform treatment-response stratification. Ji et al. integrated a PLAUR-containing immune-related signature with single-cell, spatial, and radiogenomic analyses and developed a radiotranscriptomic signature associated with immunochemotherapy response. Functional experiments from the same study further provided preclinical support for uPAR-directed intervention (47). These findings illustrate how spatially informed multimodal profiling can connect response-associated molecular features with experimentally testable therapeutic targets.

Taken together, current spatial studies support a model of iCCA as a set of interacting niches whose cellular composition and functional programs vary with anatomical context. Because most available datasets are cross-sectional, current spatial maps are better interpreted as snapshots of context-dependent organization than as direct reconstructions of temporal evolution. **Figure 1** summarizes this spatial model.

**Figure 1:**
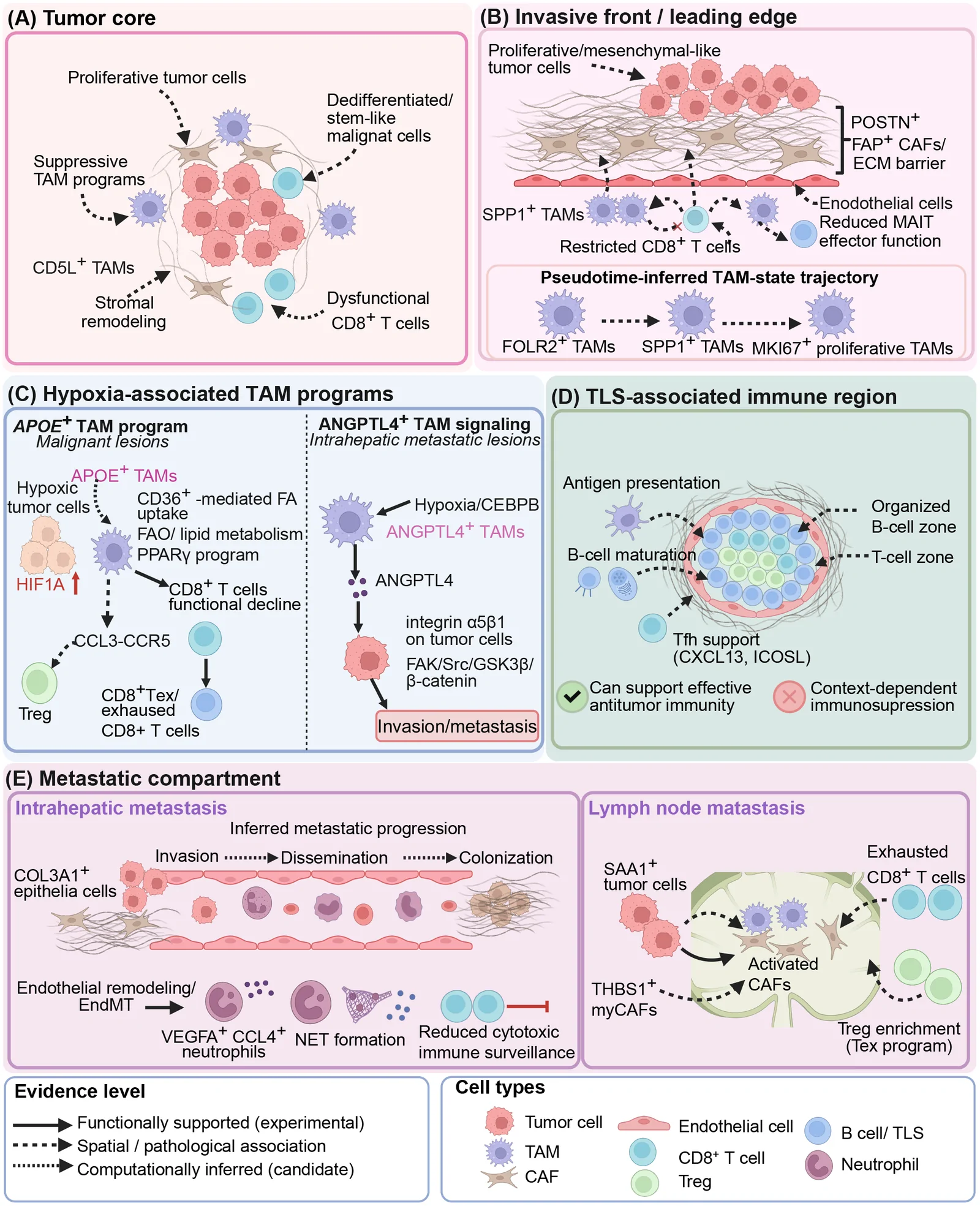
Spatially organized tumor ecosystem in intrahepatic cholangiocarcinoma. The schematic summarizes the major spatial and cellular compartments of the iCCA tumor ecosystem based on single-cell and spatial studies. **(A)** The tumor core contains proliferative and dedifferentiated tumor cell programs, suppressive TAM states, stromal remodeling, and dysfunctional CD8^+^ T cells. **(B)** At the invasive front, tumor cells are spatially associated with POSTN^+^ FAP^+^ CAFs, SPP1^+^ TAMs, and endothelial cells. These cells form an ECM-rich interface associated with restricted CD8^+^ T-cell access and reduced cytotoxic activity. **(C)** Hypoxia-associated TAM programs are context dependent. APOE^+^ TAMs are associated with immune-metabolic remodeling, Treg enrichment, and CD8^+^ T-cell dysfunction in malignant lesions, whereas ANGPTL4^+^ TAM signaling is associated with tumor-cell invasion and metastasis in intrahepatic metastatic lesions. **(D)** TLS-associated immune regions contain organized B-cell and T-cell compartments, Tfh support, antigen presentation, and B-cell maturation, reflecting context-dependent adaptive immune organization across intratumoral and peritumoral settings. **(E)** Intrahepatic and lymph-node metastases show distinct patterns of immune-stromal remodeling. These include COL3A1^+^ tumor cells, endothelial remodeling, neutrophil- and NET-associated changes, SAA1^+^ tumor cells, CD36^+^ TAMs, THBS1^+^ myCAFs, exhausted CD8^+^ T cells, and Tregs. Solid, dashed, and dotted lines indicate functionally supported relationships, spatial or pathological associations, and computationally inferred relationships, respectively. Dotted lines include candidate ligand–receptor interactions and pseudotime-inferred state trajectories. iCCA, intrahepatic cholangiocarcinoma; TAM, tumor-associated macrophage; CAF, cancer-associated fibroblast; ECM, extracellular matrix; FAO, fatty acid oxidation; MAIT, mucosal-associated invariant T cell; EndMT, endothelial-to-mesenchymal transition; TLS, tertiary lymphoid structure; Tfh, T follicular helper cell; Tregs, regulatory T cells; NET, neutrophil extracellular trap; myCAFs, myofibroblastic CAFs. Created in BioRender. YAN, Y. (2026) https://BioRender.com/5rl0dhj.

## 4 Metabolic reprogramming and immune-metabolic crosstalk in iCCA

Metabolic reprogramming adds an important layer to immune ecosystem regulation in iCCA. Metabolic states are dynamic and can be shaped by nutrient availability, hypoxia, inflammatory signaling, oncogenic pathways, pathogen-associated stimuli, and interactions with surrounding stromal and immune cells (48). In iCCA, metabolic remodeling operates at several interconnected biological levels. Intertumoral metabolic states are associated with distinct immune contextures, while metabolic dependencies intrinsic to tumor cells support cellular fitness and therapeutic adaptation. Metabolic products released by tumor cells can also directly reshape macrophage and T-cell function. Etiology-specific mechanisms constitute a further layer, as illustrated by pathogen-associated metabolic remodeling. Together, these layers clarify how metabolic programs contribute to immune heterogeneity and treatment vulnerability in iCCA.

### 4.1 Intertumoral metabolic states shape immune contexture

Integrated multi-omics profiling has classified iCCA into metabolically distinct subtypes that differ in immune composition and clinical outcome. This subtype classification links tumor-level metabolic organization with immune cell distribution, providing a useful entry point for understanding metabolic heterogeneity in the iCCA immune microenvironment.

The S1 subtype is characterized by relatively active fatty acid oxidation and fatty acid degradation programs. It is associated with a more immune-active contexture, including increased infiltration of effector-memory CD8^+^ T cells, dendritic cells, and Tregs (49). Clinically, this subtype is associated with better survival. These associations suggest that lipid-catabolic tumor states may coexist with a microenvironment capable of sustaining immune cell infiltration and immune engagement.

The S2 subtype shows a glycolysis-dominant profile, with increased expression of glycolytic enzymes such as HK1 and PFKM and reduced fatty acid degradation activity (49). This subtype is associated with an immunosuppressive contexture, including increased CD39/CD73-associated adenosine signaling, reduced effector-memory CD8^+^ T-cell infiltration, and enrichment of naive-like CD8^+^ T cells. These findings are consistent with the broader concept that glycolysis-dominant tumor states can coincide with impaired antitumor immunity. In iCCA, the S2 phenotype supports a model linking glycolytic activity, adenosine-associated immune suppression, and reduced effector T-cell infiltration. These features define a metabolically distinct, immune-restricted tumor state. The S3 subtype is characterized by a generally lower abundance of metabolism-related proteins, without dominant enrichment of a specific metabolic pathway (49). This subtype corresponds to an immune-desert-like phenotype with limited immune cell infiltration and intermediate clinical outcomes. The S1–S3 framework indicates that metabolic states in iCCA are associated with distinct immune architectures, ranging from immune-active to immune-suppressed or immune-desert-like contexts. This metabolic classification is therefore most useful for stratifying tumors according to metabolic state and immune contexture, rather than as a stand-alone explanation for immune activation or exclusion.

### 4.2 Tumor-cell-intrinsic metabolic dependencies

Beyond subtype-level metabolic differences, iCCA cells maintain specific metabolic dependencies that support proliferation, invasion, and treatment adaptation. These intrinsic metabolic programs define vulnerabilities within tumor cells and may indirectly influence the surrounding immune ecosystem. Such effects may involve altered nutrient use, cellular stress responses, and communication between tumor and stromal compartments.

MAL2 is a lipid-metabolic regulator in iCCA. Huang et al. identified MAL2 as an upstream regulator of lipogenic reprogramming. MAL2 stabilizes EGFR at the cell membrane and activates EGFR/SREBP-1 signaling, thereby increasing the expression of lipogenic enzymes, including FASN, SCD, and ACLY (50). Overexpression or knockdown of MAL2 altered the sensitivity of iCCA cells to cisplatin, supporting a role for MAL2 in cisplatin resistance in these cell lines. In xenograft models, MAL2-high tumors showed greater lipid accumulation and faster tumor growth. MAL2 is therefore linked to an intrinsic lipid-metabolic dependency that supports malignant behavior and experimental chemotherapy adaptation. Its relationship with clinical cisplatin response in patient cohorts remains to be defined.

Beyond lipid metabolism, preclinical studies have identified *de novo* glutamine synthesis as another tumor-cell-intrinsic metabolic dependency in iCCA. Zhang et al. identified SIRT6, an NAD^+^-dependent deacetylase, as an oncogenic regulator of the SIRT6–GLUL axis (51). SIRT6 enhanced the transcription of GLUL, which encodes glutamine synthetase, and increased GLUL protein stability, thereby elevating intracellular glutamine and sustaining nucleotide and amino acid pools required for tumor-cell growth. Inhibition of SIRT6 or GLUL suppressed tumor growth and enhanced the efficacy of gemcitabine–cisplatin in preclinical iCCA models. Together, MAL2-mediated lipogenesis and SIRT6–GLUL-mediated glutamine synthesis illustrate distinct tumor-cell-intrinsic metabolic adaptations: the former supports invasion, growth, and experimental cisplatin resistance, whereas the latter sustains proliferative fitness and reduces sensitivity to chemotherapy.

### 4.3 Tumor-derived metabolic signals mediate immune-metabolic crosstalk

Some metabolic programs extend beyond tumor cell fitness and directly influence immune cell behavior. In these settings, tumor-derived lipids and lipid-metabolic pathways act as intercellular signals that reshape macrophage polarization, T-cell function, and local immune surveillance.

In clinical cholangiocarcinoma specimens, Chen et al. identified a USP1-associated lipophagy–lipogenesis program and examined its mechanistic role in iCCA cell lines and mouse models (52). USP1-driven lipogenic reprogramming increased the release of long-chain unsaturated fatty acids from tumor cells. TAMs took up these lipids in a FABP5-dependent manner, leading to lipid-droplet accumulation, enhanced oxidative metabolism, reactive oxygen species generation, and an immunoregulatory macrophage state *in vitro*. Loss-of-function and rescue experiments further supported the dependence of these effects on macrophage FABP5. FABP5 knockdown reduced fatty-acid uptake and lipid-mediated macrophage polarization, whereas oleic-acid supplementation or FABP5 overexpression restored these phenotypes after USP1 depletion in tumor cells. In mouse models of iCCA, genetic deletion of USP1 in tumor cells reduced the abundance of FABP5^+^ TAMs and altered macrophage functional states (52).

This USP1-associated lipid program also affected T-cell function through macrophage-mediated mechanisms. Conditioned-medium and coculture experiments showed that TAMs conditioned by the USP1-dependent lipid program suppressed activated CD8^+^ T cell proliferation and IFN-γ production. Cell–cell communication analysis additionally implicated CCL4- and MHC-I-associated interactions between FABP5^+^ macrophages and cytotoxic T cells (52). These predicted interactions were not independently validated as causal mediators; the functional assays support the broader macrophage-to-T-cell effect rather than these specific signaling routes. The available data support a functionally validated lipid-remodeling axis from tumor cells to macrophages with downstream effects on CD8^+^ T cell activity, while the specific molecular routes linking FABP5^+^ TAMs to T-cell dysfunction remain unresolved.

### 4.4 Etiology-specific metabolic remodeling: the *Clonorchis sinensis*–FASN axis

In *Clonorchis sinensis* (*C. sinensis*)-associated iCCA, pathogen-related signals intersect with tumor lipid metabolism. Ren et al. showed that *C. sinensis* excretory-secretory products upregulated E2F1, increased FASN expression, enhanced fatty acid synthesis, and promoted malignant progression (53). This study defines a parasite-associated metabolic mechanism intrinsic to tumor cells. In a complementary study, Xu et al. demonstrated that FASN-mediated fatty acid biosynthesis remodels the immune environment in *C. sinensis*-associated iCCA (54). Increased FASN activity and free fatty acid production were associated with TAM enrichment, impaired T-cell function, and closer organization of tumor cells and macrophages. FASN inhibition reduced features of immune suppression and enhanced anti-PD-1 efficacy in *C. sinensis*-associated iCCA models (54). Together, these complementary studies define a *C. sinensis*–FASN–lipid program spanning tumor-cell-intrinsic metabolic activation and immune remodeling. Parasite-associated signals promote lipid synthesis in tumor cells, while FASN-driven lipid remodeling shapes macrophage-rich immunosuppressive niches. The available evidence is confined to *C. sinensis*-associated patient cohorts and experimental models, and these findings cannot yet be generalized to non-*C. sinensis*-associated iCCA.

### 4.5 Integrated metabolic-immune model in iCCA

Metabolic remodeling in iCCA operates across interconnected but biologically distinct layers. At the intertumoral level, metabolic subtypes define immune contextures ranging from immune-active to immune-suppressed or immune-desert-like states. Within tumor cells, MAL2-driven lipogenesis and SIRT6–GLUL-mediated glutamine synthesis support tumor growth, metabolic adaptation, and treatment resistance. At the interface between metabolism and immunity, the USP1–FABP5 lipid circuit links tumor-cell lipid release to macrophage remodeling and impaired CD8^+^ T-cell function. The *C. sinensis*–FASN axis provides an etiology-specific example in which pathogen-associated signals couple tumor-cell lipogenesis to macrophage-rich immunosuppressive niches. Although these mechanisms functionally converge on tumor adaptation and immune-ecosystem remodeling, they arise from distinct biological contexts and do not constitute a universal pathway across iCCA.

These metabolic layers intersect with the spatial immune ecosystems described above. Hypoxia, stromal organization, and inflammatory signaling can shape metabolic states, while metabolic products released by tumor cells can reprogram macrophages and alter cytotoxic lymphocyte activity. Metabolism in iCCA is therefore not solely tumor-cell intrinsic. It is an organizing component of the tumor ecosystem that links nutrient use, tumor cell plasticity, immune regulation, and therapeutic vulnerability.

Future studies combining spatial metabolomics, single-cell profiling, isotope tracing, and functional perturbation will help define metabolic program evolution within specific tumor niches. They will also clarify how these programs interact with immune cell states during therapy. Understanding these immune-metabolic relationships may support more rational combination strategies, including metabolic targeting with immunotherapy or chemotherapy in selected iCCA contexts. The progression from single-cell and spatial discovery through functional validation to biomarker development and clinical translation is summarized in **Figure 2**.

**Figure 2:**
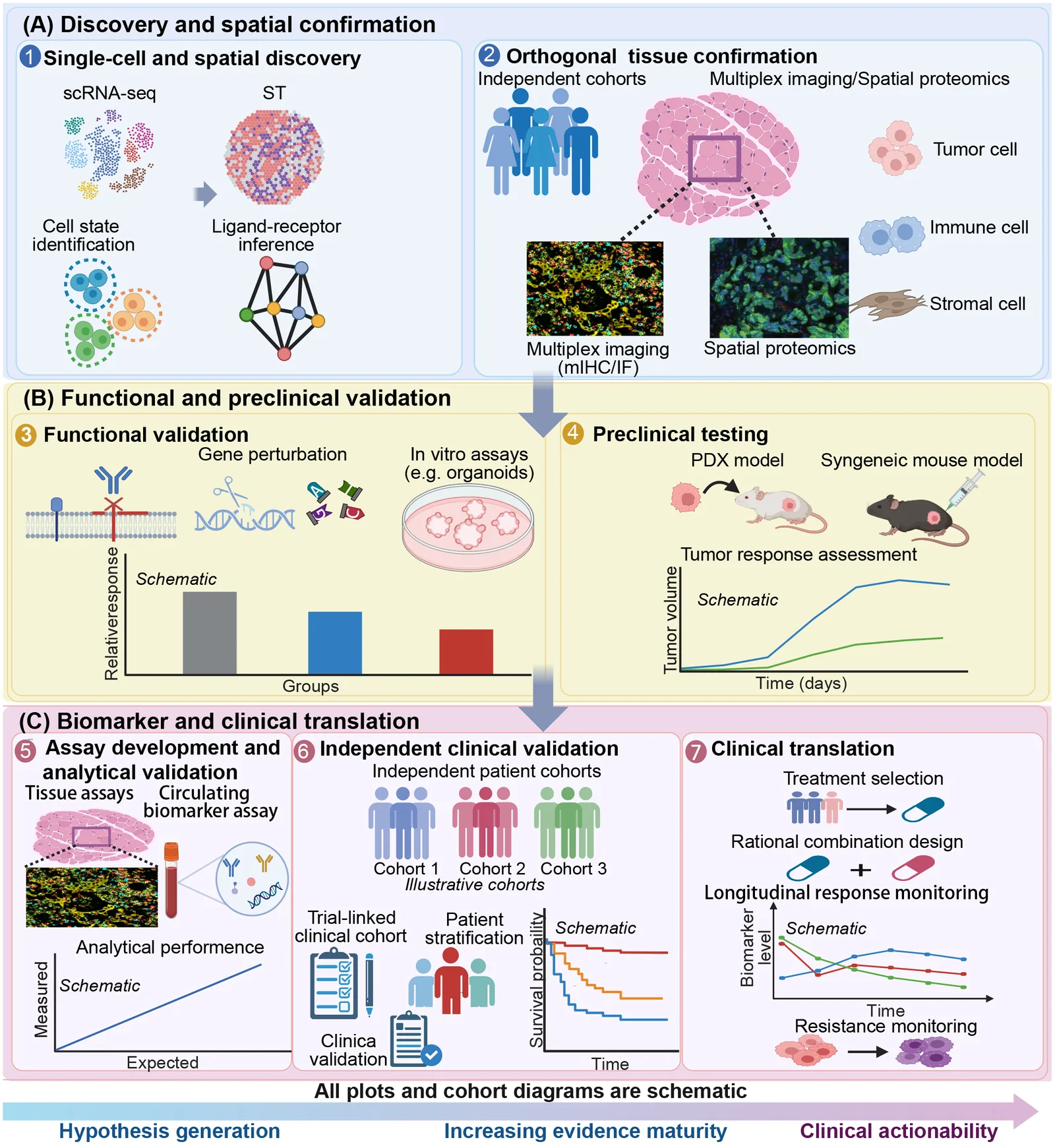
Evidence-to-translation framework for single-cell and spatial ecosystem findings in iCCA. **(A)** Discovery and spatial confirmation. Single-cell RNA sequencing and spatial transcriptomics identify candidate cell states, spatial niches, and interaction programs. Their tissue context can be further established through orthogonal confirmation in independent tissue cohorts using approaches such as multiplex imaging and spatial proteomics. **(B)** Functional and preclinical validation. Candidate mechanisms are examined through perturbation experiments, including receptor or pathway blockade, genetic manipulation, and *in vitro* functional assays, followed by preclinical testing in models such as patient-derived xenografts and syngeneic mouse models. These layers of evidence help determine whether spatially associated features are linked to reproducible biological effects. **(C)** Biomarker and clinical translation. Candidate ecosystem features can be converted into measurable biomarkers through assay development and analytical validation, followed by independent evaluation in patient cohorts or trial-linked specimens. As evidence matures, validated biomarkers may support patient stratification, treatment selection, longitudinal response assessment, and the identification of resistance-associated changes. The bottom arrow represents increasing translational readiness, evidence maturity, and clinical actionability from hypothesis generation to clinical translation. All internal plots, cohort diagrams, and quantitative-style elements are schematic or illustrative and do not represent study-derived numerical data. scRNA-seq, single-cell RNA sequencing; ST, spatial transcriptomics; mIHC/IF, multiplex immunohistochemistry/immunofluorescence; PDX, patient-derived xenograft. Created in BioRender. YAN, Y. (2026) https://biorender.com/ue9jzjf.

## 5 Translating single-cell and spatial ecosystem insights into immunotherapeutic strategies for iCCA

Translation of single-cell and spatial ecosystem findings into clinically meaningful biomarkers and therapeutic strategies requires evidence to accumulate across successive stages of validation, from spatial confirmation and functional testing to preclinical evaluation, assay development, and independent clinical validation (**Figures 2A–C**). Within this progression, scRNA-seq and ST are most informative when they connect cellular states, spatial niches, and interaction programs with reproducible biological mechanisms and clinically measurable features associated with immune suppression, treatment response, and therapeutic vulnerability.

### 5.1 Spatially informed candidate targets for immunotherapeutic development in iCCA

Alongside the metabolic vulnerabilities described above, spatially resolved immune niches have revealed candidate intervention points that address immune exclusion, myeloid suppression, and treatment-response heterogeneity. scRNA-seq and ST further define how these candidates are embedded within specific cellular neighborhoods and functional resistance states. SPP1, MARCO, and PLAUR/uPAR illustrate three complementary translational routes: disrupting an immune barrier at the invasive front, reprogramming a macrophage-centered suppressive niche, and integrating treatment-response stratification with mechanism-guided targeting, respectively. These candidates differ substantially in evidentiary maturity, ranging from hypothesis-generating spatial associations to functional perturbation and preclinical testing.

#### 5.1.1 SPP1 and immune exclusion at the invasive front

SPP1 (osteopontin) is a secreted ligand, whereas SPP1^+^ TAMs refer to a macrophage state characterized by high SPP1 expression within a broader transcriptional program. At the iCCA invasive front, SPP1^+^ TAMs are enriched in leading-edge regions containing POSTN^+^ FAP^+^ CAFs and endothelial cells, where coordinated stromal and myeloid remodeling is associated with reduced cytotoxic immune activity (34). These findings place the state of SPP1^+^ TAMs within a spatially organized stromal–myeloid program associated with immune exclusion at the tumor margin.

Preclinical HCC studies provide complementary functional evidence for a role of SPP1 signaling in immune exclusion. SPP1 blockade or loss of macrophage-derived Spp1 disrupted the SPP1^+^ macrophage–CAF barrier, increased cytotoxic T-cell infiltration, and enhanced anti-PD-1 efficacy (55). Macrophage-derived SPP1 has also been linked to impaired CD8^+^ T-cell function through SPP1–CD44 signaling (56, 57). Comparable functional evidence in iCCA remains limited. Direct inhibition of SPP1 signaling and reprogramming of SPP1^+^ TAMs should therefore be considered related but distinct therapeutic strategies.

#### 5.1.2 MARCO and suppressive niches centered on macrophages

MARCO has a different evidentiary profile as a macrophage-centered therapeutic candidate. Spatial profiling initially identified the colocalization of MARCO^+^ TAMs with CTSE^+^ tumor cells in primary iCCA, defining macrophage-enriched regions associated with reduced local immune activity, metastatic behavior, and poor prognosis. Computationally inferred galectin-related signaling, including the LGALS9–CD44 axis, further suggested that MARCO^+^ TAMs participate in a tumor–myeloid niche. This niche integrates tumor cell adaptation with local immunoregulation in these regions (43).

More recent functional evidence has extended this observation beyond spatial association. Agirre-Lizaso et al. showed that MARCO contributes to cholangiocarcinogenesis through immunosuppressive remodeling. Genetic or antibody-based interference with MARCO reduced tumor growth in mouse models of iCCA (58). Thus, the evidence supporting MARCO has progressed from spatial and prognostic association to preclinical functional perturbation. By linking spatial localization with functional perturbation, MARCO provides a stronger preclinical basis for macrophage-directed intervention than axes supported mainly by inferred communication patterns. Whether MARCO targeting can enhance checkpoint blockade remains to be evaluated clinically. However, convergent spatial and functional evidence supports evaluating this axis in combination strategies designed to remodel suppressive myeloid states.

#### 5.1.3 PLAUR–uPAR links response stratification with preclinical target validation

The PLAUR–uPAR axis follows a translational trajectory distinct from targets nominated primarily through spatial niche architecture. Ji et al. identified PLAUR as one component of a three-gene immune-related score together with CD40LG and FGFR4 and subsequently integrated single-cell, spatial, and radiogenomic analyses to characterize its tissue and immune context (47). The resulting radiotranscriptomic signature was associated with immunochemotherapy response and correlated with PLAUR expression and macrophage infiltration in tumor regions. These findings place PLAUR within a broader response-associated multimodal framework, while prospective validation is required to establish the predictive value of this signature.

The same study extended this association through functional perturbation of uPAR, the protein encoded by PLAUR. Genetic and antibody-based interference with uPAR reduced iCCA cell proliferation and migration and affected macrophage recruitment, while anti-uPAR treatment reduced tumor growth in humanized mice bearing uPAR-positive patient-derived xenografts (47). Combining anti-uPAR treatment with PD-1 blockade further enhanced antitumor activity. Together, these findings link response-associated multimodal profiling with preclinical uPAR-directed intervention, extending response stratification toward functional and preclinical evaluation.

Taken together, these candidates occupy distinct positions along the translational continuum. The therapeutic relevance of SPP1-directed intervention remains hypothesis-generating in iCCA, supported primarily by the spatial association of SPP1^+^ TAMs with an invasive-front immune-exclusion program and by functional studies of SPP1 signaling in HCC models. MARCO has stronger iCCA-specific preclinical support, linking spatial localization with genetic and antibody-based perturbation, whereas PLAUR/uPAR currently provides the most integrated biomarker-to-intervention framework through multimodal response stratification and humanized patient-derived xenograft testing. The PLAUR-associated signature and uPAR-directed strategy require independent and prospective validation. Current evidence supports prioritizing MARCO and uPAR for further iCCA-specific validation, with uPAR additionally warranting prospective biomarker assessment, whereas SPP1 requires direct functional evaluation in iCCA before therapeutic development.

### 5.2 Optimization of immunotherapeutic regimens through ecosystem-level insights

The clinical development of iCCA immunotherapy has shifted from checkpoint-inhibitor monotherapy toward combinations designed to overcome multiple ecosystem-level barriers, including myeloid suppression, stromal exclusion, abnormal vasculature, and tumor cell adaptation. However, these regimens occupy distinct stages of clinical development. TOPAZ-1 and KEYNOTE-966 establish chemotherapy plus PD-1/PD-L1 blockade as a phase III-supported first-line platform for advanced biliary tract cancer (BTC), including iCCA. Other strategies remain investigational to varying degrees, including gemcitabine–oxaliplatin plus lenvatinib and an anti-PD-1 antibody (GOLP); cryoablation plus sintilimab and lenvatinib (CASTLE); oncolytic virotherapy; metabolic-immunotherapy combinations; and intensified checkpoint strategies (59–62). scRNA-seq and ST are therefore most informative when used to define response-associated cell states and resistance mechanisms rather than to rank biologically distinct regimens on a single efficacy scale. **Figure 3** summarizes representative remodeling patterns of the iCCA immune ecosystem across dose-optimized chemoimmunotherapy, GOLP, CASTLE, and mechanism-guided metabolic or oncolytic strategies. It highlights distinct changes in myeloid states, CD8^+^ T cell trajectories, antigen presentation, and stromal, vascular, and immune-metabolic compartments.

**Figure 3:**
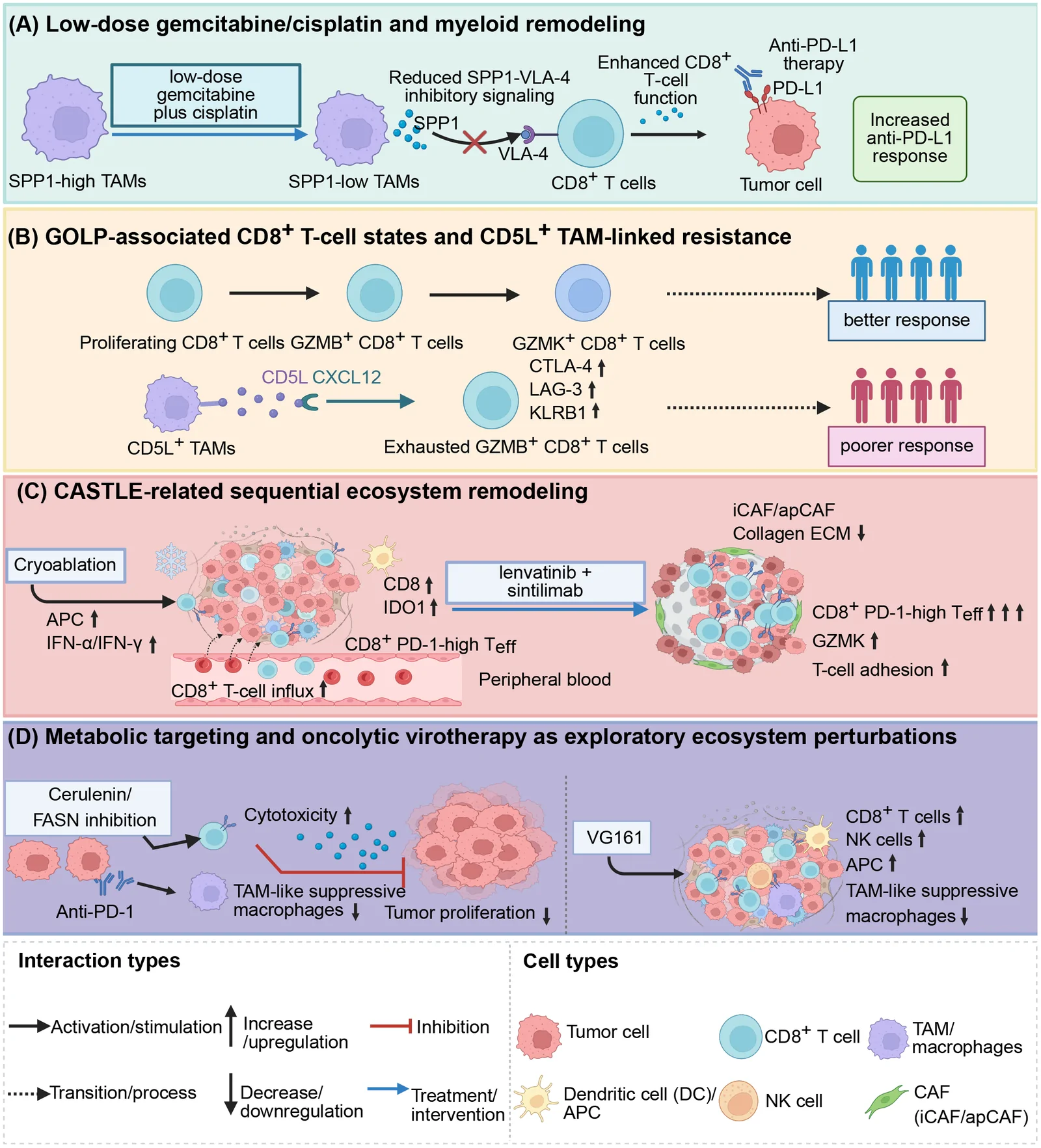
Treatment-associated remodeling of the iCCA immune ecosystem. **(A)** Low-dose gemcitabine/cisplatin combined with anti-PD-L1 therapy may remodel the myeloid compartment by reducing the abundance of SPP1^+^ TAMs and lowering SPP1 expression within TAMs, thereby weakening SPP1–VLA-4-mediated suppression of CD8^+^ T-cell infiltration and cytotoxicity. **(B)** GOLP-associated single-cell analyses link proliferating and GZMB^+^/GZMK^+^ CD8^+^ T-cell states with better response. By contrast, enrichment of CD5L^+^ TAMs and exhaustion-associated features in CD8^+^ GZMB^+^ T cells are associated with poorer response. **(C)** CASTLE illustrates a sequential remodeling model initiated by cryoablation. Cryoablation may increase tumor immunogenicity, antigen presentation, interferon-associated activation, and infiltration of CD8^+^ effector T cells with high PD-1 expression. This initial immune-activation phase is followed by remodeling of stromal, vascular, and T cell compartments mediated by sintilimab plus lenvatinib. **(D)** Exploratory ecosystem-directed strategies include FASN inhibition combined with anti-PD-1 therapy in **(C)** sinensis-associated iCCA models and, separately, oncolytic virotherapy with VG161. These approaches may enhance cytotoxic lymphocyte activity, antigen presentation, or macrophage remodeling while reducing tumor proliferation or TAM-like suppressive features. APC, antigen-presenting cell; CASTLE, cryoablation plus sintilimab and lenvatinib; DC, dendritic cell; ECM, extracellular matrix; FASN, fatty acid synthase; GOLP, gemcitabine–oxaliplatin plus lenvatinib and an anti-PD-1 antibody; iCCA, intrahepatic cholangiocarcinoma; iCAF, inflammatory cancer-associated fibroblast; apCAF, antigen-presenting cancer-associated fibroblast; LDGC, low-dose gemcitabine/cisplatin; NK, natural killer cell; TAM, tumor-associated macrophage; Teff, effector T cell; VLA-4, very late antigen-4. Created in BioRender. YAN, Y. (2026) https://biorender.com/rsiroi9.

#### 5.2.1 From phase III chemoimmunotherapy standards to dose-optimized immune remodeling

TOPAZ-1 and KEYNOTE-966 provide independent phase III evidence that adding PD-1/PD-L1 blockade to gemcitabine and cisplatin (GemCis) can improve survival in advanced BTC, including iCCA. In TOPAZ-1, durvalumab plus GemCis improved overall and progression-free survival. The objective response rate increased to 26.7%, compared with 18.7% with chemotherapy alone, without a substantial increase in grade 3–4 adverse events (60). KEYNOTE-966 independently showed an overall-survival benefit with pembrolizumab plus GemCis in the overall BTC population, with a consistent effect in the prespecified intrahepatic subgroup. However, the overall objective response rate was approximately 29% in both treatment groups (62). These phase III trials establish chemotherapy-checkpoint blockade as a therapeutic platform rather than a regimen-specific observation. Response rates were modest, and KEYNOTE-966 showed no advantage in objective response rate. The dissociation between survival benefit and objective response suggests that the effect of checkpoint blockade may not be fully explained by greater initial tumor shrinkage, leaving room for biological optimization.

Single-cell analyses have raised a complementary question: whether chemotherapy dose and schedule can be optimized not only for cytotoxicity but also for immune remodeling. Liang et al. compared conventional-dose and low-dose gemcitabine/cisplatin regimens, each combined with PD-1/PD-L1 blockade, in mouse models of iCCA. The low-dose strategy preserved antitumor activity, reduced the abundance of immunosuppressive SPP1^+^ TAMs, and reprogrammed STAT1/STAT3 signaling within TAMs, thereby lowering SPP1 expression and weakening SPP1–VLA-4-mediated suppression of CD8^+^ T cells (63). The study also reported preliminary clinical observations consistent with activity and tolerability, but these data remain exploratory and require prospective confirmation.

The importance of this study lies less in establishing a new clinical standard than in reframing chemotherapy as an immunomodulatory component whose dose may influence the tumor ecosystem. The balance between SPP1^+^ TAMs and effector CD8^+^ T cells could serve as a correlative endpoint in future trials. Such trials could test whether dose-dependent immune remodeling is reproducible and translates into survival or tolerability benefits relative to phase III-validated chemoimmunotherapy. In this context, dose optimization is best viewed as a mechanistically informed refinement of an established chemoimmunotherapy platform. **Figure 3A** summarizes these treatment-associated changes in myeloid and T-cell states.

#### 5.2.2 GOLP: integrating chemotherapy, antiangiogenic targeting, and PD-1 blockade

The GOLP regimen combines gemcitabine-oxaliplatin, lenvatinib, and PD-1 blockade and therefore targets tumor cell proliferation, vascular signaling, and immune suppression concurrently. A single-center phase II study evaluated toripalimab plus lenvatinib and gemcitabine-oxaliplatin (GEMOX) in 30 patients with advanced iCCA. The objective response rate was 80%, with median progression-free and overall survival of 10.2 and 22.5 months, respectively. Grade 3 or higher adverse events occurred in 56.7% of patients (64). These results were encouraging but were derived from a small, nonrandomized cohort. More recently, the randomized phase II–III neoGOLP trial evaluated neoadjuvant GOLP in resectable high-risk iCCA. Median event-free survival was 18.0 months with neoadjuvant GOLP versus 8.7 months with surgery-first management. These randomized data provide substantially stronger evidence that the multidimensional strategy can improve outcomes in a defined perioperative population (61). Phase III evidence for the advanced-disease regimen remains pending (NCT05342194).

The single-cell studies accompanying GOLP are particularly informative because they move beyond regimen efficacy toward response-state biology. Profiling of pretreatment and on-treatment tumors linked higher baseline proportions of proliferating CD8^+^ T cells and GZMB^+^ CD8^+^ effector cells to better response. Lower baseline abundance of CD5L^+^ TAMs was also associated with better response. Longitudinal analysis suggested that favorable responses involved transitions from proliferating and GZMB^+^ CD8^+^ states toward a GZMK^+^ state. By contrast, tumors with poor responses showed incomplete state transitions and greater T-cell dysfunction. CD5L^+^ TAMs contributed to this resistance phenotype by promoting inhibitory programs in effector T cells, and anti-CTLA-4 treatment reversed resistance in mouse models of iCCA (65).

The paired single-cell data provide a biologically plausible explanation for heterogeneous responses to GOLP. They suggest that treatment response may be associated with both the baseline composition of effector T cells and therapy-induced transitions among CD8^+^ T-cell states, with the latter shaped by suppressive TAMs (65). The candidate biomarkers were derived from exploratory single-cell analyses of pre- and post-treatment tumors. Prospective validation with standardized sampling and predefined thresholds will be important for assessing their value in treatment selection.

The IMbrave151 experience provides an important boundary for interpreting GOLP. In the randomized phase II IMbrave151 study, adding bevacizumab to atezolizumab plus GemCis did not improve median progression-free or overall survival in the iCCA subgroup (66). Differences in antiangiogenic agents, checkpoint inhibitors, chemotherapy backbones, and treatment settings limit direct comparison between IMbrave151 and GOLP. Even so, the contrast suggests that antiangiogenic-immunochemotherapy synergy is context dependent and is likely to vary with vascular, stromal, and immune states rather than follow from pathway complementarity alone. **Figure 3B** illustrates response-associated trajectories of CD8^+^ T cells and a resistance state linked to CD5L^+^ TAMs.

#### 5.2.3 CASTLE: local ablation as an initiator of systemic immune remodeling

CASTLE provides a distinct model of combination therapy in which a local intervention is used to initiate systemic immune remodeling. In the single-arm phase II CASTLE-01 trial, 28 patients with advanced or metastatic iCCA after chemotherapy received cryoablation followed by sintilimab and lenvatinib. The regimen achieved an objective response rate of 75% and a disease control rate of 100%. Median progression-free and overall survival were 16.8 and 25.4 months, respectively (59). These outcomes are notable in a previously treated population, but the small sample size and absence of a randomized comparator prevent direct efficacy comparisons with other systemic regimens.

Paired pretreatment and on-treatment multi-omics analyses from CASTLE provide an opportunity to examine temporal immune remodeling. These analyses assessed how local ablation, antiangiogenic therapy, and PD-1 blockade may reshape the iCCA immune ecosystem. Exploratory analyses supported a sequential remodeling model in which cryoablation increased tumor immunogenicity and dendritic-cell activation. Lenvatinib altered vascular and stromal states to facilitate lymphocyte infiltration, and PD-1 blockade helped maintain intratumoral effector T cell activity (59). Baseline “hot” versus “cold” transcriptional phenotypes did not clearly distinguish responders, whereas on-treatment changes in immune cell composition and spatial organization appeared more informative. This sequence suggests that physical barriers, abnormal vasculature, and insufficient immune activation may need to be modified sequentially rather than targeted in isolation. The utility of this model for treatment selection remains to be established in randomized studies. A related second-line phase II study comparing the combination of cryoablation, lenvatinib, and PD-1 blockade with FOLFOX chemotherapy is now ongoing (NCT06860477). This sequential remodeling model, spanning immune activation, stromal and vascular reorganization, and enhanced effector T-cell infiltration, is illustrated in **Figure 3C**.

#### 5.2.4 Emerging ecosystem-guided perturbation strategies

Early mechanism-guided approaches extend the analysis beyond checkpoint-centered combinations. Studies of VG161 examine whether enhanced antigen presentation and immune activation can shift selected iCCA tumors toward a more inflamed state. Multi-omics analyses accompanying preliminary clinical activity showed enhanced antigen presentation, CD8^+^ T cell activation, and macrophage remodeling (67). FASN inhibition addresses a different biological constraint. In *C. sinensis*-associated iCCA models, suppressing FASN-mediated fatty acid synthesis reduced macrophage-associated immune suppression and enhanced anti-PD-1 activity (54).

These strategies remain at an earlier stage of development than phase III chemoimmunotherapy or the clinically evaluated multidimensional regimens discussed above. Their main value is conceptual. They show how ecosystem profiling can nominate distinct biological constraints, including weak immune priming and lipid-dependent immune suppression, that can be perturbed experimentally. **Figure 3D** summarizes these two examples.

Taken together, the therapeutic studies discussed in this section support an evidence hierarchy rather than a menu of interchangeable combination strategies. Phase III chemoimmunotherapy provides the clinical anchor. Dose modulation, GOLP, and CASTLE examine how myeloid, vascular, stromal, and treatment-associated remodeling may modify response, whereas virotherapy and metabolic targeting remain earlier mechanistic extensions of the same framework. The central translational question is which ecosystem states identify tumors that are susceptible to each form of perturbation.

Longitudinal single-cell and spatial profiling may be particularly informative because treatment response is unlikely to be determined by baseline cell abundance alone. Resolving how myeloid, stromal, vascular, metabolic, and T-cell states change during therapy could help distinguish biomarkers that correlate with outcome from ecosystem features that inform treatment selection or adaptation.

## 6 Discussion

Integrated scRNA-seq and ST studies support a spatial ecosystem model in which TLS-associated adaptive regions (29–31), invasive-front stromal–myeloid interfaces (34), hypoxia-associated niches (41), and metastatic ecosystems (45, 46) organize distinct immune states. Across these compartments, macrophage and fibroblast programs vary with anatomical context (34, 42, 45, 46), while tumor metabolism reshapes immune composition and function (49, 52, 54). Together, these findings support reciprocal ecosystem remodeling rather than a single dominant pathway (44, 68). Because most datasets are cross-sectional, the identified niches are better interpreted as context-dependent states than as fixed stages of a common temporal trajectory.

This ecosystem perspective also clarifies therapeutic evidence. Phase III trials establish GemCis plus PD-1/PD-L1 blockade as a first-line platform for advanced BTC, including iCCA (60, 62), whereas response-associated signatures, candidate targets, and combination strategies remain at earlier translational stages (47, 54, 58, 59, 62–67). Their value lies in identifying the tumor–immune and stromal states in which an intervention may be biologically relevant.

The major unresolved question is therefore which ecosystem features represent reproducible properties of iCCA and which remain dependent on biological context. Many single-cell and spatial studies are based on relatively small and often single-center cohorts (**Supplementary Table 1**) and therefore incompletely represent the histologic, molecular, etiological, anatomical, and treatment-related heterogeneity of iCCA (1, 3). Etiology may contribute to this variation, as illustrated by *C. sinensis*-associated iCCA, in which parasite-related signals and FASN-dependent lipid remodeling are linked to distinct metabolic and immune phenotypes (53, 54). Anatomical site is equally important: intrahepatic and lymph-node metastases contain different tumor–immune–stromal programs (45, 46). Some features, particularly TLS-associated immune organization, are supported across independent iCCA cohorts, whereas many myeloid, metastatic, and metabolic programs remain supported mainly by single-cohort or contextually convergent evidence (**Table 1**). Reproducibility should therefore be judged at the level of conserved biological programs and spatial relationships rather than by expecting identical cell clusters or niche compositions across all studies.

**Table 1:** Evidence summary of major spatial, metabolic, and therapeutic findings in iCCA.

Major finding	Evidence status	Spatial evidence	Functional validation	Clinical relevance	Major uncertainty
Context-dependent organization of TLSs and B cells (29–32)	Cross-cohort, context-dependent support	Context-dependent organization of TLSs and B cells.	Ex vivo and preclinical studies support B-cell dysfunction and VEGF/ICB-mediated immune remodeling.	TLS features are prognostic; treatment response is associated with circulating BAFFR^+^ B cells and B-cell clonotypes, rather than TLS abundance.	Prognostic associations vary with TLS location, maturity, histologic subtype, and stromal context; predictive value remains unproven.
Invasive-front stromal–myeloid niche (33, 34, 37)	Contextual/convergent support	SPP1^+^ TAMs, POSTN^+^ FAP^+^ CAFs, and endothelial cells localize to leading-edge regions.	Direct evidence for targeting SPP1 in iCCA is limited; functional support comes mainly from broader liver-cancer models.	Provides a biological rationale for immune-exclusion biomarkers and niche remodeling.	Evidence from broader liver-cancer studies does not constitute independent replication in iCCA.
Hypoxia-associated myeloid programs (41, 42)	Contextual/convergent support	Spatial organization of APOE^+^ TAMs and Tregs in primary lesions; ANGPTL4^+^ TAMs in intrahepatic metastases.	Metabolic polarization and ANGPTL4 perturbation have functional support.	Linked to survival-associated T-cell states and serum/tissue ANGPTL4 associations.	APOE^+^ and ANGPTL4^+^ TAMs are context-specific states, not a single validated lineage.
Niches comprising MARCO^+^ TAMs and tumor cells (43, 58)	Contextual/convergent support	mIHC supports proximity of MARCO^+^ TAMs and CTSE^+^ tumor cells.	Genetic or antibody-based MARCO interference reduces tumor growth in iCCA models.	Spatial organization is associated with poor prognosis and metastatic behavior.	No clinical evidence yet for MARCO-directed therapy or ICB combinations.
Metastatic site-specific ecosystems (45, 46)	Contextual/convergent support	Distinct COL3A1^+^ epithelial programs in liver metastases and nodal niches comprising SAA1^+^ tumor cells and CD36^+^ TAMs.	Functional assays support COL3A1-associated endothelial and NET remodeling and SAA1–CD36-mediated tumor-cell invasion.	Defines candidate mechanisms of metastatic adaptation.	Specific axes arise from single site-specific cohorts and have not been replicated across metastatic sites.
Metabolic heterogeneity and intrinsic tumor metabolism (49–51)	Single-cohort/context-specific evidence	Direct spatial validation of metabolic subtypes remains limited.	MAL2 and SIRT6-GLUL dependencies have separate functional support.	Links metabolic state to immune contexture, growth, and treatment adaptation.	Subtype classification requires independent validation and spatial confirmation.
USP1–FABP5 lipid-mediated crosstalk (52)	Single-study/context-specific evidence	Tissue and model evidence supports tumor-immune crosstalk; spatial validation remains limited.	Loss/rescue and TAM–CD8 assays support USP1/FABP5 dependence and downstream immune effects; CCL4/MHC-I interactions remain inferred.	Preclinical rationale for targeting lipid-mediated immune suppression.	Single-study evidence; the pathway from FABP5^+^ TAMs to T-cell dysfunction remains unresolved.
Etiology-specific *C. sinensis*–FASN axis (53, 54)	Contextual/convergent support	Patient and model data link *C. sinensis*–FASN activity to macrophage-rich immune states.	FASN perturbation limits tumor growth and immune suppression and enhances anti-PD-1 activity in etiology-matched models.	Candidate metabolism-directed immunotherapy for *C. sinensis*-associated iCCA.	Generalizability to non-*C. sinensis*-associated iCCA has not been established.
PLAUR-containing response-associated gene signature and uPAR-directed preclinical targeting (47)	Single-cohort/context-specific evidence	Integrated radiogenomic, single-cell, and spatial analyses support associations within the tissue context.	Humanized PDX experiments support uPAR targeting, alone or in combination with PD-1 blockade.	Candidate framework linking response stratification with therapeutic development.	The response signature and uPAR-directed therapeutic strategy have not been independently or prospectively validated.
Treatment-associated ecosystem remodeling (59–67)	Contextual/convergent support	Paired profiling in GOLP and CASTLE links response to changing immune and stromal states.	Preclinical perturbation supports selected resistance mechanisms; clinical evidence varies by regimen.	Phase III chemoimmunotherapy provides the clinical platform; other strategies remain investigational.	Biomarkers are regimen-specific and require prospective validation.

Evidence-status note: Cross-cohort, context-dependent support denotes evidence from multiple iCCA cohorts without an identical finding or uniform direction of association. Contextual/convergent support denotes related but non-equivalent evidence across studies, anatomical sites, etiologies, or broader liver cancer settings. Single-cohort/context-specific evidence and single-study/context-specific evidence denote findings lacking independent replication.

BAFFR, B-cell-activating factor receptor; CAF, cancer-associated fibroblast; ECM, extracellular matrix; FASN, fatty acid synthase; GOLP, gemcitabine–oxaliplatin plus lenvatinib and an anti-PD-1 antibody; ICB, immune checkpoint blockade; iCCA, intrahepatic cholangiocarcinoma; mIHC, multiplex immunohistochemistry; NET, neutrophil extracellular trap; PDX, patient-derived xenograft; ST, spatial transcriptomics; TAMs, tumor-associated macrophages; TLS, tertiary lymphoid structure; uPAR, urokinase plasminogen activator receptor; VEGF, vascular endothelial growth factor.

Methodological robustness is a related but distinct issue. The robustness of cell-state annotation depends on the clustering strategy, reference choice, annotation framework, and extent of support across independent iCCA cohorts (6, 69, 70). Integration of single-cell and spatial data introduces further model dependence because inferred cellular composition and localization are influenced by transcriptomic coverage, spatial resolution, segmentation, reference composition, and computational strategy (12, 23–25, 71). Cross-study agreement is therefore more informative when it occurs at the level of transcriptional programs, anatomical localization, neighboring cell populations, and associated functions.

A similar evidence hierarchy applies to intercellular communication. Ligand–receptor compatibility and spatial proximity can identify plausible interactions, but their interpretation depends on the assumptions of the analytical framework (72, 73). Orthogonal imaging and spatial proteomic approaches can strengthen tissue-level localization and protein-level evidence (74), while perturbation experiments provide stronger support for functional relationships. Such evidence is available for selected iCCA mechanisms involving hypoxia-associated macrophage signaling, tumor–myeloid crosstalk, metabolic immune remodeling, and MARCO-directed intervention (41, 44, 54, 58). Computational inference, spatial association, and functional validation should therefore be viewed as complementary but non-equivalent levels of evidence.

The final challenge is determining which reproducible ecosystem features can be translated into clinically measurable biomarkers. Current studies illustrate different stages of this transition: B cell states have been associated with chemoimmunotherapy outcome (31), ANGPTL4 has been linked to tissue and circulating measurements associated with metastatic disease (41), and multimodal radiogenomic analyses have identified a PLAUR-containing signature associated with immunochemotherapy response (47). These findings highlight the need to distinguish prognostic associations and treatment-associated correlations from prospectively validated predictive biomarkers. Prospective multicenter and longitudinal studies could help determine which ecosystem features remain stable across patients and anatomical compartments, which change during treatment, and which are informative for response or resistance. Broader advances in spatial omics and noninvasive ecosystem profiling may also provide routes for translating complex tissue states into more scalable clinical readouts (71, 75, 76).

Overall, scRNA-seq and ST have established a framework for viewing iCCA as a spatially organized and metabolically regulated tumor ecosystem. The next advance is unlikely to come from cataloging additional cell populations or spatial niches alone. More important will be identifying which ecosystem features are reproduced across biological contexts, which reflect context-dependent adaptation, which are supported by functional evidence, and which can be measured robustly enough to inform therapeutic stratification.
