# Supplementary material for: Single-cell and spatial transcriptomics in intrahepatic cholangiocarcinoma: spatial immune ecosystems, metabolic remodeling, and immunotherapeutic implications
Source: Front Immunol. 2026 Oct 20;17:1918458. doi: 10.3389/fimmu.2026.1918458 (PMC13728433; doi:10.3389/fimmu.2026.1918458)
Supplement: Supplementary file 1 [file Table1.docx]

**Supplementary Table 1 Representative single-cell and spatial transcriptomic studies of the iCCA tumor ecosystem**

| **Samples (n)** | **Platform** | **Major findings** | **Key cellular populations** | **Validation/evidence level** | **Clinical relevance** | **Key limitations** | **Reference** |
| --- | --- | --- | --- | --- | --- | --- | --- |
| 7 iCCA tumors; 3 ANT specimens | scRNA-seq: 10x Genomics Chromium | Generated an early single-cell atlas of iCCA and identified reciprocal tumor–vCAF crosstalk. | Malignant epithelial cells; Tregs; TAMs; CD146⁺ vCAFs; other CAF subsets | Functional evidence supported the vCAF–IL-6/EZH2/miR-9-5p circuit; most other interaction axes were computationally inferred. | Foundational map of the tumor–immune–stromal ecosystem; identified a potential CAF-associated therapeutic vulnerability. | Modest cohort; no native ST; loss of spatial context; incomplete functional validation of inferred interactions | (35) |
| scRNA-seq: 9 patients/25 specimens, including 7 TC, 9 LE, and 9 matched ANT specimens; ST: 5 LE specimens | scRNA-seq: 10x Genomics Chromium; ST: 10x Genomics Visium | Defined a leading-edge stromal–myeloid niche associated with proliferative tumor cells and impaired CD8⁺ T-cell function. | Proliferating tumor cells; POSTN⁺FAP⁺ CAFs; endothelial cells; SPP1⁺ macrophages; MAIT cells; low-cytotoxicity CD8⁺ T cells | Integrated scRNA-seq and ST with IHC/mIHC support; MAIT-cell-mediated macrophage recruitment and macrophage-state transitions were mainly inferential. | Identified the leading edge as an immune-restrictive spatial niche and provided a rationale for targeting SPP1-associated myeloid remodeling. | Small patient cohort; leading-edge-focused spatial sampling; cross-sectional design; limited receptor-specific perturbation | (34) |
| GSE138709: 5 patients/8 treatment-naïve samples; HRA001748: 25 tumors/4 normal specimens; external ST: 21 sections, including 1 iCCA LE section | scRNA-seq: 10x Genomics Chromium (GSE138709 and HRA001748); ST: 10x Genomics Visium | Defined a hypoxia-associated immune niche linking APOE⁺ TAM metabolic remodeling and Treg enrichment with heterogeneous CD8⁺ T-cell states, including CXCL13⁺ precursor-exhausted T cells. | APOE⁺ TAMs; Tregs; CXCL13⁺ precursor-exhausted CD8⁺ T cells  terminally exhausted CD8⁺ T cells; hypoxic myeloid–Treg niche | Mechanistic evidence supported the HIF1A–PPARγ–CD36/FAO program; the APOE⁺ TAM–Treg interaction was supported mainly by spatial and correlative evidence. | Identified prognostic immune states and candidate hypoxia-associated biomarkers. | Public-data reanalysis; only one external iCCA ST section; limited receptor-specific validation; retrospective prognostic associations | (42) |
| Primary scRNA-seq: 8 iCCA tumors; public scRNA-seq: 35 tumors—GSE125449, 10; GSE138709, 5; GSE151530, 8; GSE181878, 12; ST: 6 iCCA tumor sections | scRNA-seq: 10x Genomics, primary and public datasets; ST: 10x Genomics Visium | Identified MARCO⁺ TAMs–CTSE⁺ tumor cell colocalization as a macrophage-rich immunosuppressive niche. | MARCO⁺ TAMs; CTSE⁺ tumor cells; plasma-cell-rich pattern; macrophage-rich immune-resistant niche | Spatial colocalization and clinical associations were supported; LGALS9–CD44 remained a candidate communication axis. | Identified a prognostic spatial niche and potential targets for myeloid-directed intervention. | Primary scRNA-seq and ST cohorts were not patient-matched; no immunotherapy-treated cohort; no MARCO/CTSE/LGALS9 perturbation; Visium resolution limited cell-specific interaction assignment | (43) |
| Human: 6 iCCA tumors and 3 corresponding PT specimens; mouse: 6 iCCA samples | scRNA-seq: BD Rhapsody | Defined a TAM-associated dedifferentiation–immunosuppression loop and distinguished malignant-cell dedifferentiation from proliferation. | YAP1⁺ dedifferentiated malignant cells; proliferating malignant cells; SPP1⁺ macrophages; resident macrophages; LILRB1/LILRB2⁺ TAMs | Human multi-omics data was combined with coculture experiments and macrophage-targeting mouse models; ligand-level evidence varied among modules. | Supported macrophage-directed strategies targeting tumor plasticity and immune suppression. | No ST; several interaction edges were derived from NicheNet; therapeutic implications remain preclinical | (44) |
| scRNA-seq: 16 patients/28 specimens, including 12 primary tumors with IM, 12 paired intrahepatic metastases, and 4 nonmetastatic tumors; ST: HRA000437, CNP0002199 | scRNA-seq: SeekOne; ST: 10x Genomics Visium (HRA000437) and Stereo-seq (CNP0002199) | Inferred invasion- and colonization-associated states during iCCA liver metastasis and linked COL3A1⁺ epithelial cells to endothelial remodeling and neutrophil/NET-associated metastatic colonization. | COL3A1⁺ epithelial cells; EndMT cells; VEGFA⁺CCL4⁺ neutrophils; NET-associated metastatic niche | Functional evidence supported the effects of COL3A1⁺ epithelial cells and NET-associated colonization; JAG1–NOTCH3 was identified computationally without axis-specific blockade. | Provided a metastatic-niche framework and identified candidate anti-metastatic intervention points. | Limited metastatic cohort; external ST datasets; treatment-predictive value not established; several ligand–receptor axes lacked specific perturbation | (45) |
| 4 iCCA tumors; 4 ANT specimens; 7 lymph-node specimens | scRNA-seq: 10x Genomics Chromium | Characterized a lymph-node-metastasis ecosystem involving SAA1⁺ tumor cells and CD36⁺ macrophages. | SAA1⁺ tumor cells; CD36⁺ macrophages; exhausted CD8⁺ T cells; Tregs; THBS1⁺ myCAFs | scRNA-seq was supported by mIHC/spatial-proximity analysis and in vitro evidence for SAA1/CD36-associated crosstalk. | Identified candidate lymph-node-metastasis biomarkers and potential myeloid–metabolic intervention points. | scRNA-seq cohort limited to 4 patients; lymph-node-specific context; no clinical therapeutic testing; no ST | (46) |
| scRNA-seq: 15 treatment-naïve iCCA tumors; ST subset: 6 tumors | scRNA-seq: 10x Genomics Chromium; ST: 10x Genomics Visium | Linked *C. sinensis*-associated FASN-mediated fatty-acid biosynthesis to TAM enrichment, impaired T-cell function, and macrophage-rich immunosuppressive remodeling. | FASN-high malignant cells; TAM-like macrophages; dysfunctional T cells; infection-associated immune–metabolic niche | Multicenter multi-omics, spatial, and preclinical evidence was provided; clinical predictive evidence remained associative. | Supported the rationale for combining FASN inhibition with anti-PD-1 therapy in *C. sinensis*-associated iCCA. | Etiology-specific cohort; small ST subset; immunotherapy-predictive relevance requires prospective validation | (54) |
| 10 patients/18 tumors: 8 paired pretreatment and post–3-cycle GOLP specimens (16 specimens) and 2 treatment-naïve surgical specimens | scRNA-seq and scTCR-seq: 10x Genomics Chromium | Linked enrichment of CD5L⁺ TAMs to GZMB⁺ CD8⁺ T-cell exhaustion and impaired response to GOLP. | CD5L⁺ TAMs; GZMB⁺ CD8⁺ T cells; proliferating CD8⁺ T cells; GZMK⁺ CD8⁺ T cells; CTLA-4-associated exhausted T cells | Paired treatment scRNA-seq/scTCR-seq analyses were combined with mouse anti-CTLA-4 rescue experiments; human biomarker evidence remained small-cohort and post hoc. | Identified response-associated immune states and supported a potential CTLA-4-based resistance-reversal strategy. | Small cohort receiving a single regimen; only 8 longitudinally paired patients; no ST; predictive thresholds were not prospectively validated | (65) |
| GSE171899: 6 iCCA tumors with paired ANT specimens | scRNA-seq: 10x Genomics Chromium (GSE171899) | Defined compartment-dependent B-cell biology linking mature TLS/peritumoral B cells with favorable prognosis and tumor-infiltrating B-cell dysfunction. | BAFFR⁺ B cells; tumor-infiltrating B cells; mature TLS B-cell regions; immunoregulatory B cells; BCR clonotypes | Pathology and flow cytometry were combined with single-cell reanalysis and ex vivo cytokine-blockade experiments; response biomarkers remained associative. | Mature TLS-associated B-cell organization was linked to prognosis, whereas circulating BAFFR⁺ B cells and expanded B-cell clonotypes were associated with chemoimmunotherapy response; predictive value requires prospective validation. | Low intratumoral B-cell frequency; limited spatial and subset resolution; use of peripheral or healthy-donor B-cell assays; prospective validation required | (31) |
| 15 patients: IM, 8; non-IM, 7 | scRNA-seq: 10x Genomics Chromium | Identified a hypoxia-induced ANGPTL4⁺ TAM program that promoted intrahepatic metastasis through macrophage–tumor signaling. | ANGPTL4⁺ TAMs; CEBPB⁺ macrophages; hypoxia-responsive epithelial cells; metastatic malignant cells | Mechanistic and preclinical evidence supported the ANGPTL4 macrophage–tumor axis; serum biomarker evidence remained associative. | Identified ANGPTL4 as a candidate serum biomarker and therapeutic target for intrahepatic metastasis. | Small biopsy cohort; no ST; serum biomarker performance and therapeutic targeting require independent prospective validation | (41) |

Note: Representative studies were selected to cover major published scRNA-seq and/or ST analyses of iCCA that inform malignant-cell heterogeneity, immune and stromal remodeling, spatial niches, metastatic ecosystems, immune–metabolic interactions, treatment-associated immune states, and translational relevance. Only the modalities performed or analyzed in each study are listed. Sample numbers refer to patients, specimens, or tissue sections as specified. Public or external datasets are distinguished from study-generated datasets. Reference numbers correspond to the reference list of the main manuscript.

Abbreviations: ANT, adjacent non-tumor tissue; BCR, B-cell receptor; CAF, cancer-associated fibroblast; EndMT, endothelial-to-mesenchymal transition; FAO, fatty acid oxidation; GOLP, gemcitabine–oxaliplatin plus lenvatinib and an anti-PD-1 antibody; IHC, immunohistochemistry; IM, intrahepatic metastasis; LE, leading edge; MAIT, mucosal-associated invariant T cell; mIHC, multiplex immunohistochemistry; NET, neutrophil extracellular trap; non-IM, non-intrahepatic metastasis; PT, peritumoral tissue; scRNA-seq, single-cell RNA sequencing; scTCR-seq, single-cell T-cell receptor sequencing; ST, spatial transcriptomics; TAMs, tumor-associated macrophages; TC, tumor core; TLS, tertiary lymphoid structure; Tregs, regulatory T cells; vCAF, vascular cancer-associated fibroblast; PreTex, precursor-exhausted T cell; Tex, terminally exhausted T cell.
